# Association between Psoriasis Vulgaris and Coronary Heart Disease in a Hospital-Based Population in Japan

**DOI:** 10.1371/journal.pone.0149316

**Published:** 2016-02-24

**Authors:** Masayuki Shiba, Takao Kato, Moritoshi Funasako, Eisaku Nakane, Shoichi Miyamoto, Toshiaki Izumi, Tetsuya Haruna, Moriaki Inoko

**Affiliations:** 1 Department of Cardiovascular Medicine, Hyogo Prefectural Amagasaki General Medical Center, Amagasaki, Hyogo, Japan; 2 Cardiovascular Center, Tazuke Kofukai Medical Research Institute, Kitano Hospital, Osaka, Japan; 3 Department of Cardiovascular Medicine, Graduate School of Medicine, Kyoto University, Kyoto, Japan; Osaka University Graduate School of Medicine, JAPAN

## Abstract

**Background:**

Psoriasis vulgaris is a chronic inflammatory skin disease with an immune-genetic background. It has been reported as an independent risk factor for coronary heart disease (CHD) in the United States and Europe. The purpose of this study was to investigate the association between psoriasis and CHD in a hospital-based population in Japan.

**Methods:**

For 113,065 in-hospital and clinic patients at our institution between January 1, 2011 and January 1, 2013, the diagnostic International Classification of Diseases (ICD)-10 codes for CHD, hypertension, dyslipidemia, diabetes, and psoriasis vulgaris were extracted using the medical accounting system and electronic medical record, and were analyzed.

**Results:**

The prevalence of CHD (n = 5,167, 4.5%), hypertension (n = 16,476, 14.5%), dyslipidemia (n = 9,236, 8.1%), diabetes mellitus (n = 11,555, 10.2%), and psoriasis vulgaris (n = 1,197, 1.1%) were identified. The prevalence of CHD in patients with hypertension, dyslipidemia, diabetes, and psoriasis vulgaris were 21.3%, 22.2%, 21.1%, and 9.0%, respectively. In 1,197 psoriasis patients, those with CHD were older, more likely to be male, and had more number of the diseases surveyed by ICD-10 codes. Multivariate analysis showed that psoriasis vulgaris was an independent associated factor for CHD (adjusted odds ratio [OR]: 1.27; 95% confidence interval [CI]: 1.01–1.58; p = 0.0404) along with hypertension (adjusted OR: 7.78; 95% CI: 7.25–8.36; p < 0.0001), dyslipidemia (adjusted OR: 2.35; 95% CI: 2.19–2.52; p < 0.0001), and diabetes (adjusted OR: 2.86; 95% CI: 2.67–3.06; p < 0.0001).

**Conclusion:**

Psoriasis vulgaris was independently associated with CHD in a hospital-based population in Japan.

## Introduction

Psoriasis vulgaris is a chronic inflammatory skin disease with an immune-genetic background. Although its pathogenesis is not fully understood, there is solid evidence of a susceptibility locus in the human leukocyte antigen region [1, 2]. Genetic polymorphisms in the vascular endothelial growth factor (VEGF) gene may also contribute to psoriasis and differ between Caucasians and Asians [3]. Psoriasis is common in Caucasians but is rare in Africans [4, 5]. In Asians, psoriasis is less common. The prevalence of psoriasis vulgaris is 0.34% of the general population in Japan [6]. This prevalence is lower than that of the general population in the United States and Europe (2.0–4.0%) [7–9].

Systemic low-grade inflammation may be involved in the pathogenesis of psoriasis [10, 11]. Patients with psoriasis vulgaris typically have a higher prevalence of dyslipidemia, hypertension, smoking, and obesity compared to those without psoriasis [12]. These comorbidities overlap with the risk factors of coronary heart disease (CHD) [12–15], although the precise mediated mechanisms linking psoriasis and CHD have not been clear.

Many studies in the United States and Europe have shown that psoriasis is an independent risk factor for CHD and cardiovascular events [16–24] or cardiovascular mortality [25]; however, in contrast, other studies concluded that that psoriasis is not an independent risk factor for coronary atherosclerosis and acute cardiovascular events [26–30]. These conflicting results may be because of the differences in the study design, severity of psoriasis, or confounders and effect modifiers, such as psoriatic arthritis [31]. In Japan, the relationship between psoriasis vulgaris and CHD has almost never been studied. One reason for this is the rarity of psoriasis vulgaris in Japanese patients [6]. Considering that an immune-genetic background strongly affects the prevalence of psoriasis and the common underlying causes such as systemic low-grade inflammation, the association between psoriasis and CHD should also be apparent in Japanese patients. The aims of this study were to investigate the relationship between psoriasis vulgaris and CHD in a hospital-based population in Japan.

## Methods

### Patient population

The medical accounting system and electronic medical record (EMR) data of all clinic and in-hospital patients of all ages at our institution between January 1, 2011 and January 1, 2013 were studied retrospectively. From the EMR data, we extracted patients’ data, including their name, age, and sex by each disease, i.e., psoriasis vulgaris (International Classification of Diseases [ICD]-10 code L40.0), hypertension (ICD-10 codes I10, I11, I12, I13, I14, and I15), dyslipidemia (ICD-10 code E78), diabetes mellitus (ICD-10 codes E10, E11, E12, E13, and E14), and CHD. CHD included ischemic heart disease, myocardial infarction (acute, sub-acute, or old, i.e., ICD-10 codes I21, I22, and I25.2, respectively), and angina pectoris (I20). Each disease was regarded as present when the diagnoses were recorded in the hospital charts. The total number of in-hospital and clinic patients at our institution was determined by the medical accounting system. The research protocol was approved by the institutional review board of Kitano Hospital according to the ethical guidelines of the 1975 Declaration of Helsinki (P15-06-005). Since this is a retrospective study, the consent was not obtained and patient records/information was anonymized and de-identified prior to analysis.

### Statistics

Continuous variables are expressed as the mean ± standard deviation. Mean numbers of risk factors are expressed as the mean and 95% confidence interval (CI). In comparisons of the baseline characteristics of the study population, the chi square test was used for categorical variables and the Wilcoxon rank sum test was used for continuous variables when appropriate. Differences in categorical variables among two or more groups were analyzed using the Cochran-Mantel-Haenszel test. Associations between the diseases surveyed and CHD in patients overall were analyzed using a multivariate logistic regression model, which was adjusted for hypertension, dyslipidemia, diabetes mellitus, and psoriasis. In subgroup analysis of patients who had at least one disease, including CHD, hypertension, dyslipidemia, diabetes, and psoriasis, the association between factors were analyzed by using a multivariate logistic regression model, which was adjusted for hypertension, dyslipidemia, diabetes mellitus, psoriasis, and sex as categorical variables and age as a continuous variable. Adjusted odds ratios (ORs) and 95% CIs were calculated. A p value <0.05 was considered statistically significant in all analyses. Statistical analyses were performed using JMP software, version 10 (SAS Corp.).

## Results

The prevalence of CHD (n = 5,167, 4.2%), hypertension (n = 16,476, 14.5%), dyslipidemia (n = 9,236, 8.1%), diabetes mellitus (n = 11,555, 10.2%), and psoriasis vulgaris (n = 1,197, 1.0%) were identified (Table 1). The prevalence of CHD in patients with hypertension, dyslipidemia, diabetes, and psoriasis vulgaris were 21.3% (3,510/16,476), 22.2% (2,056/9,236), 21.1% (2,442/11,555), and 9.0% (108/1197), respectively (Table 1).

**Table 1:** Factors related to coronary heart disease (CHD) among the study patients.

		CHD		
	Total	Present	Absent
Variables	(n = 113,065)	(n = 5167)	(n = 107,898)
**With hypertension**	16,476 (14.5%)	3,510 (21.3%)	12,966 (78.7%)
Mean age (SD)	70.7 (14.3)	75.6 (11.0)	69.4 (14.8)
Male (n, %)	8,642 (52.4%)	2,165 (61.8%)	6,477 (49.9%)
**With dyslipidemia**	9,236 (8.1%)	2,056 (22.2%)	7,180 (77.7%)
Mean age (SD)	70.0 (3.4)	75.3 (10.6)	68.5 (13.7)
Male (n, %)	4,293 (46.1%)	1,211 (58.9%)	3,082 (42.9)
**With diabetes mellitus**	11,555 (10.2%)	2,442 (21.1%)	9,113 (78.9%)
Mean age (SD)	69.7 (13.2)	74.9 (10.3)	68.3 (13.5)
Male (n, %)	6,741 (58.3%)	1,602 (65.6	5,139 (56.3)
**With psoriasis**	1,197 (1.0%)	108 (9.0%)	1,089 (91.0%)
Mean age (SD)	64.9 (18.1)	75.0 (12.8)	63.1 (19.0)
Male (n, %)	762 (63.6%)	81 (75.0%)	681 (62.5%)
**With CHD**	5,167 (4.2%)		
Mean age (SD)	74.0 (12.0)		
Male (n, %)	3,059 (59.2%)		

Regardless of whether patients did or did not have psoriasis, patients with CHD had more number of the three major diseases (hypertension, dyslipidemia, and diabetes) related to CHD than those without CHD (p < 0.001, Table 2). In 1,197 psoriasis patients, those with CHD were older and more likely to be male than those without CHD (p < 0.001), and they had the highest number of the above-mentioned diseases than the other groups (p < 0.001 by the Cochran-Mantel-Haenszel test, Table 2).

**Table 2:** Comparison of the characteristics in psoriasis patients with and without CHD.

	With psoriasis			Without psoriasis		
		CHD				CHD		
	Total	Present	Absent	Total	Present	Absent
Variable	(n = 1,197)	(n = 108)	(n = 1,089)	(n = 111,868)	(n = 5,059)	(n = 106,809)
**Age (y.o., mean, SD)**	64.1 ± 18.9	75.0 ± 12.8	63.1 ± 19.0	n/a	74.0 ± 12.0	n/a
**Male (n, %)**	762 (63.6%)	81 (75%)	681 (62.5%)	n/a	2978 (58.8%)	n/a
**Hypertension**	288 (24.0%)	81 (75.0%)	207 (19.0%)	16,188 (14.4%)	3,429 (67.7%)	12,759 (11.9%)
**Dyslipidemia**	231 (19.2%)	63 (58.3%)	168 (15.4%)	9,005 (8.0%)	1,993 (39.3%)	7,012 (6.5%)
**Diabetes mellitus**	252 (21.0%)	60 (55.5%)	192 (17.6%)	11,303 (10.1%)	2,382 (47.0%)	8,921 (8.3%)
**No. of the three major diseases**^#^						
**3**	73 (6.1%)	27 (25.0%)	46 (4.2%)	2,883 (2.5%)	1,066 (21.0%)	1,817 (1.7%)
**2**	173 (14.5%)	49 (45.4%)	124 (11.4%)	7,112 (6.3%)	1,594 (31.5%)	5,518 (5.1%)
**1**	204 (17.0%)	25 (23.1%)	179 (16.5%)	13,623 (12.1%)	1,418 (28.0%)	12,205 (11.4%)
**0**	747 (62.3%)	7 (6.5%)	740 (68.0%)	88,250 (78.8%)	981 (19.3%)	87,269 (81.7%)
**Mean No. of the disease**^#^ **(95% CI)**	0.644 (0.590–0.697)	1.888 (1.725–2.052)	0.520 (0.469–0.571)	0.326 (0.322–0.330)	1.542 (1.524–1.560)	0.268 (0.264–0.272)

^#^: hypertension, dyslipidemia, and diabetes mellitus, CI: confidence interval, n/a: not available.

**Fig 1:**
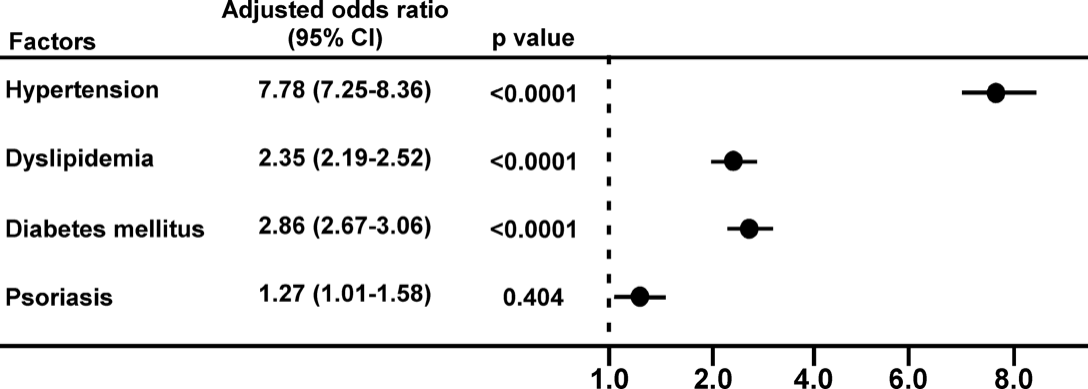
Multivariate logistic regression analysis of the factors related to coronary heart disease. Data are adjusted for hypertension, dyslipidemia, diabetes mellitus, and psoriasis.

Multivariate logistic regression analysis after adjusting for hypertension, dyslipidemia, and diabetes, and psoriasis, showed that psoriasis vulgaris was still independently associated with CHD (adjusted OR: 1.27; 95% CI: 1.01–1.58; p = 0.0404), along with hypertension (adjusted OR: 7.78; 95% CI: 7.25–8.36; p < 0.0001), dyslipidemia (adjusted OR: 2.35; 95% CI: 2.19–2.52; p < 0.0001) and diabetes (adjusted OR: 2.86; 95% CI: 2.67–3.06; p < 0.0001) in an entire hospital-based population (Fig 1). We next assessed 25,799 patients who have at least one disease including CHD, hypertension, dyslipidemia, diabetes, and psoriasis (Table A in S1 File). The psoriasis also shows an independent association with CHD after adjustment for sex and age (Table B in S1 File) in these patients.

## Discussion

In this retrospective study, we showed the prevalence of psoriasis vulgaris in a hospital-based population in Japan. We also determined that psoriasis vulgaris is independently associated with CHD in these patients.

Psoriasis has been associated with chronic systemic inflammation mediated by the type 1 helper T (Th1) cell [32, 33] and Th17 cell [10, 34, 35]. The Th1 cell stimulates macrophages that secrete tumor necrosis factor α (TNF-α). Recently, there has been an increasing interest in the Th 17 cell pathway [36] and related pro-inflammatory cytokines in psoriasis, including interleukin (IL)-17 and IL23 [37], which induce the production of inflammatory mediators such as IL1, IL6, IL8, and TNF-α [33]. TNF-α activates endothelium and facilitates leukocyte invasion into surrounding tissues, resulting in the formation of infiltrated plaques, and causes insulin resistance in endothelial cells through the p38 mitogen-activated protein kinase pathway [38]. Chronic inflammation is associated with insulin resistance, which leads to endothelial dysfunction by disturbing vasodilation through the endothelial nitric oxide synthase pathway [39]. The contribution of genetic polymorphisms to the VEGF gene in psoriasis has also been reported [3]. These data support the association between psoriasis and CHD through chronic low-grade systemic inflammation and endothelial dysfunction via the overlapping risks for CHD, as also shown in Japanese patients in the present study.

Psoriasis vulgaris has been reported to be independent risk factors for CHD in Europe and United States [16–24]. However, a recently published study by Parisi (2015), including a cohort of 48,523 patients with psoriasis and 208,187 controls and a greater number of predictor variables, did not find an association between psoriasis (hazard ratio [HR]: 1.02, 95%CI: 0.96–1.08) or severe psoriasis (HR:1.29, 95%CI: 0.97–1.70) and the risk for cardiovascular events [30]. In contrast, Dregan et al. (2014), using the same database and inception cohort design, found an independent association between psoriasis and coronary heart disease; their point estimate of the HR of coronary heart disease in severe psoriasis was 1.29 (95% CI: 1.01–1.64, p = 0.042). [23]. Ogdie also reported, using similar database, that the risk of cardiovascular events was higher in patients with psoriasis not prescribed a disease modifying anti-rheumatic drug (HR 1.08, 95%CI: 1.02–1.15) and in patients with severe psoriasis (disease modifying anti-rheumatic drug users: HR 1.42, 95%CI: 1.17–1.73) [40]. One reason for these conflicting results may be the inclusion of psoriatic arthritis as an independent confounder in the Parisi study [30,31]. Psoriatic arthritis is associated with psoriasis vulgaris; hence, it can be considered an effect modifying factor that moderates the relationship between psoriasis and outcome [31]. When psoriatic arthritis excluded, the fully adjusted HR of psoriasis and severe psoriasis was 1.46 (95%CI: 1.11–1.92) [30]. A possible association between severe psoriasis and cardiovascular disease was reported by a systematic review of 14 epidemiologic studies [41]. However, whether cardiovascular disease is directly associated with psoriasis remains uncertain, as many of these studies, as well as our present study, could not exclude the possibility of inadequate adjustment for risk factors [41].

Another reason for this inconsistency may be the study design (inception or prevalent cohort). An inception cohort fully captures risk in case of early disease-related outcomes. However, in the setting of psoriasis and CV risk, disease duration (and thus long-term exposure to inflammation) is related to the outcome, with increased risk of the poor outcome as the duration increases. [17]. Thus, use of an inception cohort with short-term follow-up will result in underestimation of the true effect. The prevalent cohort, in contrast, better represents psoriasis patients in the general population. Prevalent designs, however, also have limitations. [31]. The prevalent designs may lead to underestimation of the effect through exclusion of outcomes related to mortality (as in the cases of death from cardiovascular events). [23,40]. Our study design is cross-sectional with one time-point observation. The cardiovascular outcomes were not evaluated but the presence of CHD was assessed with psoriasis in the present study; hence we only addressed the independent association (adjusted OR: 1.27; 95% CI: 1.01–1.58; p = 0.0404).

There have been reports that the long-term use of anti-hypertensive drugs and hypertension itself are possibly associated with the development of psoriasis vulgaris [15,39,42]. Dyslipidemia has been also closely associated with psoriasis [43], although the mechanism of connections between psoriasis vulgaris and dyslipidemia is not clear. In another report which analyzed Japanese psoriasis patients, the prevalence of hyperlipidemia and hypertension ranged from 16.7% to 20.7% and from 22.8 to 28.5%, respectively [6], which is consistent with the prevalence in our analysis (Table 2). Table 3 shows the ORs of CHD reported previously in general or hospital-based population in Japan and other countries [17, 44–46]. ORs for CHD in this study were comparable for diabetes mellitus and dyslipidemia but seemed to be high for hypertension compared to those of the general population. The prevalence of CHD in patients with psoriasis was 9% (95% CI: 7.38–10.62) in the present analysis. The prevalence of CHD in the entire hospital-based population was 4.5% (95%CI: 4.44–4.69) in the present analysis, whereas the prevalence of CHD in general population estimated by the government data was 0.71% (95%CI: 95%CI: 0.66–0.76) which is calculated from the estimated total numbers of CHD patients (911,000) and the estimated numbers of total population (127, 435,000) in Japan [47]. Thus, several sort of bias may exist in the hospital-based population in the present study. Although further population-based studies are required for generalization, we showed that psoriasis vulgaris is an independent associated factor for having CHD in the entire hospital-based population in the present study (Fig 1) and in patients with one or more disease codes surveyed after adjustment of age and sex (Table B in S1 File).

**Table 3:** Reported odds ratios (95% confidence intervals) of risk factors for coronary heart disease.

		Odds ratio (95% confidence interval)		
Source	Population	Hypertension	Dyslipidemia	Diabetes mellitus
Kawano H[44]	Hospital based population (Japan)	4.8 (3.8–5.95)	1.28 (1.00–1.62)	3.44 (2.50–4.75)
Iso H[45]	General population (male, Japan)	2.1 (1.1–3.8)	2.5 (1.5–4.3)	1.0 (0.5–0.9)
Iso H[45]	General population (female, Japan)	1.3 (0.6–2.8)	1.8 (0.9–3.4)	0.5 (0.1–2.1)
Armstrong AW[17]	Hospital based population (US)	1.55 (1.36–1.77)	1.87 (1.66–2.12)	1.63 (1.42–1.88)
Medrano MJ[46]	General population (Spain)	1.24 (0.88–1.73)	1.97 (1.42–2.73)	1.52 (1.0–2.33)
Shiba M (this study)	Hospital based population (Japan)	7.78 (7.25–8.36)	2.35 (2.19–2.52)	2.86 (2.67–3.06)

### Study Limitations

There were several limitations in the study. First, our study did not consider patients’ smoking status or blood tests. Smoking is a risk factor of psoriasis vulgaris [13]. Smoking may affect both CHD and psoriasis. Therefore, smoking would be an important factor. However, it was difficult to determine the smoking habits of all psoriasis patients because the EMR data were retrospective and disease-specific. In addition, since the data of the EMR of the total patients (113,065) was too large to be processed and the total number of patients was collected from the medical accounting system instead, the age and sex of the entire cohort were unknown. It is possible that other unknown confounding factors, including body weight and genetic backgrounds, affected the result. Lack of these data might lead to an inadequate adjustment. In addition, our study population did not represent the general Japanese population. Second, diagnostic codes consistent with the diseases may have included registered codes acceptable to the Japanese health care insurance system. There is a possibility that we overestimated or underestimated the prevalence of these diseases. Third, our study did not demonstrate a temporal relationship or dose-responsiveness. Finally, the CI of psoriasis was relatively broad (95%CI: 1.01–1.58). Therefore, we could not conclude that psoriasis is a risk factor of CHD. Patients with a longer duration of psoriasis vulgaris (defined as >8 years) tended to have CHD [17]. Psoriasis disease characteristics and medications may also impact the clinical outcome of CHD in those with psoriasis [10, 48]. Substantial undertreatment of coronary risk factors is noted in patients with severe psoriasis by Danish nationwide registry [49]. It is controversial whether biological drugs for psoriasis vulgaris, e.g., TNF-α inhibitors and IL12/23 inhibitors, reduce the risk for cardiovascular events [50–52]. Further population-based studies that consider psoriasis characteristics/treatment, age, sex, smoking, etc. are required to generalize and verify that psoriasis is an independent risk factor for CHD in the Japanese population.

## Conclusion

Psoriasis vulgaris was independently associated with CHD in a hospital-based population in Japan.

## Supporting Information

S1 File Analysis of patients who have at least one disease including CHD, hypertension, dyslipidemia, diabetes, and psoriasis. Characteristics (Table A) and a multivariate logistic regression analysis (Table B).
(DOCX)
