# Correction: Association between Psoriasis Vulgaris and Coronary Heart Disease in a Hospital-Based Population in Japan

**DOI:** 10.1371/journal.pone.0158699

**Published:** 2016-06-29

**Authors:** Masayuki Shiba, Takao Kato, Moritoshi Funasako, Eisaku Nakane, Shoichi Miyamoto, Toshiaki Izumi, Tetsuya Haruna, Moriaki Inoko

Fig 1 appears incorrectly in the published article. Please see the correct Fig 1 and its caption below.

**Fig 1:**
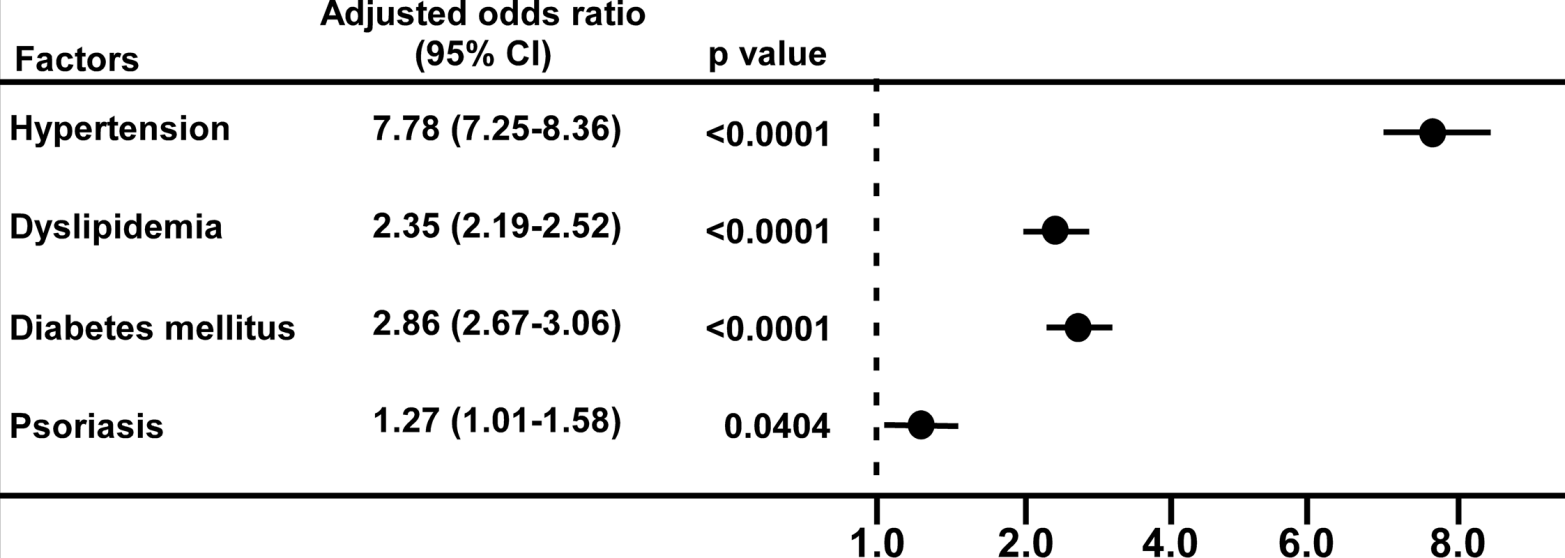
Multivariate logistic regression analysis of the factors related to coronary heart disease. Data are adjusted for hypertension, dyslipidemia, diabetes mellitus, and psoriasis.
