# Supplementary material for: Association between Psoriasis Vulgaris and Coronary Heart Disease in a Hospital-Based Population in Japan
Source: PLoS One. 2016 Feb 24;11(2):e0149316. doi: 10.1371/journal.pone.0149316 (PMC4766013; doi:10.1371/journal.pone.0149316)
Supplement: S1 File — Characteristics (Table A) and a multivariate logistic regression analysis (Table B). (DOCX) [file pone.0149316.s001.docx]

**Table A. Characteristics of patients who have at least one disease including CHD, hypertension, dyslipidemia, diabetes, and psoriasis**

|  | **Subgroup**  **total** |  |  |  |  |  |
| --- | --- | --- | --- | --- | --- | --- |
|  |  | **CHD** | **Hypertension** | **Dyslipidemia** | **DM** | **PV** |
| **Variables** | (n = 25,799) | (n = 5,167) | (n = 16,476) | (n = 9,236) | (n = 11,555) | (n = 1,197) |
| **Mean age (SD)** | 68.9 (14.8) | 74.0 (12.0) | 70.7 (14.3) | 70.0 (3.4) | 69.7 (13.2) | 64.9 (18.1) |
| **Male (n, %)** | 13,318 (51.6%) | 3,059 (59.2%) | 8,642 (52.4%) | 4,293 (46.1%) | 6,741 (58.3%) | 762 (63.6%) |
| **No. of risk factors^#^** |  |  |  |  |  |  |
| **3** | 2,956 (11.4%) | 1,093 (21.1%) | 7,369 (44.7%) | 2,956 (32.0%) | 2,956 (34.2%) | 73 (6.1%) |
| **2** | 7,286 (28.2%) | 1,643 (31.7%) | 6,151 (37.3%) | 3,774 (40.8%) | 4,647 (40.2%) | 173 (14.5%) |
| **1** | 13,827 (53.5%) | 1,443 (27.9%) | 7,369 (44.7%) | 2,506 (27.1%) | 3,952 (34.2%) | 204 (17.0%) |
| **0** | 1,730 (6.7%) | 988 (19.1%) |  |  |  | 747 (62.3%) |

^#:^ hypertension, dyslipidemia, and diabetes mellitus.

CHD: coronary heart disease, DM: diabetes mellitus, PV: psoriasis vulgaris.

The data of age and sex in hypertension, dyspipidemia, Diabetes, and PV was also provided in Table 1. The number of risk factors in PV was also provided in Table 2.

**Table B.** **Multivariate logistic regression analysis of the factors related to coronary heart disease in patients who have at least one disease including CHD, hypertension, dyslipidemia, diabetes, and psoriasis**

| **Factors** |  | **Adjusted OR (95% CI)** | **p value** |
| --- | --- | --- | --- |
| **Hypertension** | Adjustment 1 | 1.23 (1.15–1.31) | <0.0001 |
|  | Adjustment 2 | 1.01 (0.94–1.08) | 0.6554 |
| **Dyslipidemia** | Adjustment 1 | 1.23 (1.15–1.31) | <0.0001 |
|  | Adjustment 2 | 1.22 (1.14–1.31) | <0.0001 |
| **Diabetes mellitus** | Adjustment 1 | 1.14 (1.06–1.21) | <0.0001 |
|  | Adjustment 2 | 1.01 (0.95–1.08) | 0.5521 |
| **Psoriasis** | Adjustment 1 | 2.25 (1.84–2.78) | <0.0001 |
|  | Adjustment 2 | 2.49 (2.04–3.08) | <0.0001 |

Adjustment 1: Data are adjusted for hypertension, dyslipidemia, diabetes mellitus, and psoriasis. Adjustment 2: Data are adjusted for hypertension, dyslipidemia, diabetes mellitus, psoriasis, sex, and age. OR: odds ratio, CI: confidence interval
